# Determination of the Fatty Acid Profile and Lipid Quality Indices in Selected Infant Formulas

**DOI:** 10.3390/molecules29092044

**Published:** 2024-04-29

**Authors:** Aleksandra Purkiewicz, Renata Pietrzak-Fiećko

**Affiliations:** Department of Commodity Science and Food Analysis, Faculty of Food Science, University of Warmia and Mazury in Olsztyn, Plac Cieszynski 1, 10-719 Olsztyn, Poland; renap@uwm.edu.pl

**Keywords:** food quality, lipid profile, milk for infants, human milk

## Abstract

The quality of fat in infant milk is determined by the fatty acid profile and selected indices describing nutritional value. The aim of this study was to analyze the fatty acid profile and lipid quality indices of infant formulas and compare these data with breast milk. The study material included seven types of cow’s milk-based follow-on infant formulas and samples of mature breast milk. The determination of fatty acids was performed using the gas chromatography (GC) technique. Lipid quality indices were calculated based on the relevant equations. Infant formulas contained more medium-chain fatty acids (MCFAs) and oleic acid. Moreover, they contained more than 30% more linoleic acid and more than twice as much α-linolenic acid and docosahexaenoic acid. In contrast, significant amounts of trans fatty acids (TFAs) were noted in breast milk, while infant formulas contained trace amounts. Infant formulas were characterized by a lower AI (Index of Atherogenicity) (0.49–0.98) and TI (Index of Thrombogenicity) (0.48–0.60) and a higher H/H (hypocholesterolemic/hypercholesterolemic) ratio (1.93–2.30) compared with breast milk (1.47, 1.60, and 1.21, respectively). The composition of infant formulas depended on the type of fat added at the production stage and differed significantly from breast milk, particularly in terms of polyunsaturated fatty acids and lipid quality indices.

## 1. Introduction

In infant nutrition, the most recommended method of feeding that contributes to the balanced development of the child is natural feeding. Among the advantages of breast milk are its nutritionally optimized composition and the widely understood psychological aspect, defined as the formation of a mother–child bond during feeding [1]. Children fed with breast milk are more likely to have full intellectual potential resulting in better performance in adulthood. Moreover, breastfeeding women reach their pre-pregnancy weight faster and are at lower risk of breast and ovarian cancers [2]. However, there are some medical and physiological contraindications to natural feeding, such as poor mammary gland development or infection/inflammation, maternal infection with HIV, herpes simplex, herpes zoster, hepatitis C, or hormonal imbalance. In such situations, a recommended and safe alternative is the use of infant formulas with a composition analogous to breast milk [3]. 

The consumption of commercial infant formulas is widespread worldwide. Their contribution to infant feeding is a partial or complete replacement for breast milk in infant and young child nutrition [4]. Infant formulas can be divided into three main categories: first-feeding infant formula (0–6 months), follow-on infant formula (6–12 months), and infant formulas for young children (13–36 months). These products are usually based on cow’s milk and lactose, with the addition of vegetable oils (sunflower, canola, palm, coconut, soybean), vitamins, and minerals [5]. Infant formulas are available on the market in three forms: liquid, powder, and ready-to-eat. Among these, the most popular and cheapest form is powder, the appropriate amount of which must be mixed with water [6]. 

Lipids constitute an average of 3–5% of the composition of breast milk and infant formulas. They provide important functions in the child’s body, including the formation of the cell membrane, proper maturation and functioning of the nervous system, and regulating cognitive and motor development during the first year of the child’s life [7]. In addition, they provide more than 50% of energy, including essential fatty acids (FAs) and fat-soluble vitamins, thus meeting the child’s nutritional needs [8]. It has been reported that the composition of breast milk is extremely complex and variable depending on, among other things, the lactation period or the mother’s diet [9]. The lipid composition of breast milk is mainly composed of triacylglycerols (TGs) (98%), with the remainder consisting of phospholipids, cholesterol, non-esterified fatty acids, and mono- and diacylglycerols [9]. In contrast, the fat in infant formulas used nowadays is based on a mixture of vegetable oils; hence, their composition is much less complex than that of breast milk. Nevertheless, monounsaturated oils, fractionated lipids, or phospholipids from eggs and fish oils are also added as a source of fat in infant formulas currently produced. These fats are added primarily to improve the quality of the lipid fraction of infant formulas [9]. It has been reported that mature breast milk has a composition of about 34–47% saturated fatty acids (SFAs), mainly palmitic (17–25%), 31–43% monounsaturated fatty acids (MUFAs), up to 26% *n*-6 PUFAs, and up to 3.6% *n*-3 PUFAs. The situation is different for infant formulas, where the composition of fatty acids depends on the lipid source used. The use of palm oil translates into a high content of palmitic acid, while coconut oil increases the proportion of short- and medium-chain acids in infant formulas [10]. Infant formulas provide all the necessary nutrients for normal growth and development to newborns [7]. However, despite technological advances, there are still large differences in the lipid composition of infant formulas and breast milk. Plant oils added to infant formulas contain oleic and linoleic acid; however, they do not ensure the presence of α-linolenic acid and long-chain polyunsaturated fatty acids [3].

The lipid quality of a particular food product is assessed through a detailed analysis of the fatty acid profile. In addition, the correlations and ratios of individual fatty acids, including desirable fatty acids (DFAs), hypocholesterolemic/hypercholesterolemic (H/H) ratio, Atherogenicity Index (AI), and Thrombogenicity Index (TI) [11], are used to assess the impact of a particular product on consumer health. Infant formulas are an excellent source of nutrients when natural feeding is not possible for certain reasons. There are many studies in the literature characterizing the quality of breast milk lipids [7,8,12,13,14]. However, there are still only a few works comparing infant formulas in terms of lipid profile and fat quality. Moreover, few authors have evaluated lipid quality indices in infant milk. The aim of this study was (1) to evaluate the fatty acid profile of available follow-on infant formulas based on cow’s milk, (2) to estimate lipid quality indices in infant formulas, and (3) to collate and compare the data obtained to mature breast milk.

## 2. Results and Discussion

### 2.1. Comparison of Fatty Acid Profile in Infant Formulas from Different Manufacturers

The data presented in Table 1 show the content of each fatty acid group. Twenty different fatty acids were identified in the various infant formulas, which included seven from the saturated fatty acid (SFA) group, three from the monounsaturated fatty acid (MUFA) group, five from the *n*-3 and *n*-6 polyunsaturated fatty acid (PUFA *n*-3, PUFA *n*-6) group, and five from the trans fatty acid (TFA) group.

#### 2.1.1. Saturated Fatty Acids (SFAs)

In each of the infant formulas studied, SFA [38.28–45.16%] and MUFA [35.34–40.33%] predominated. A study by Wu et al. [15] found similar results in one of the infant formulas studied [41.28% SFA and 36.94% MUFA]. In contrast, the other infant formula studied by the authors had more MUFA [46.34%] than SFA [32.07%]. The main reason for the different proportions of the different fatty acid groups is the use of different fat sources for the production of infant formulas. Infant formulas may have different sources of fat added in different proportions, including sunflower, canola, coke, palm, or fish [3]. 

Among medium-chain fatty acids (MCFAs), three acids were detected in each of the infant formulas: caprylic (C8:0), capric (C10:0), and lauric (C12:0). IF-III was the richest in MCFA [17.03%], which, relative to the other formulas, contained the highest percentages of these individual three acids (*p* ≤ 0.05). The MCFA content of IF-VI [8.14%] and IF-VII [7.55%] was lower relative to IF-III by more than 50% and 55%, respectively. MCFAs are reported to be extremely important for infant growth and development; these acids are characterized by more efficient and faster absorption compared with long-chain fatty acids (LCFAs). The most prevalent MCFA in the infant formulas tested was lauric acid, with percentages ranging from 6.40% to 12.86% in the seven different formulas. Similar results regarding the proportion of MCFAs were obtained by Wu et al. [15]. In contrast, Mendonça et al. [3] indicated lauric acid levels of 5.95% in standard infant formula, representing significantly lower values than those determined in our study (*p* ≤ 0.05). In a study by Mendonça et al. [3], lauric acid levels were higher in specialized infant milk (preterm infant formulas, hypoallergenic formulas). In the conducted study, among the SFAs, each of the infant formulas was richest in palmitic acid (C16:0), with levels ranging from 17.85% in IF-III to 26.17% in IF-VII. As for the total SFA content of infant formulas, according to the literature, it ranges from 28.70% to 48.99% of total fatty acids [3], and the SFA content of each of the infant formulas tested fell within the range stated.

#### 2.1.2. Monounsaturated Fatty Acids (MUFAs)

The level of MUFA in the tested formulas ranged from 35.46% in IF-I to 40.33% in IF-VII. The content of myristoleic (C14:1 *n*-5) and palmitoleic (C16:1 *n*-7) particular infant formulas was significantly lower than that determined by Wu et al. [15]. The predominant acid of the MUFA group in the milk studied was oleic acid (C18:1 *n*-9). In the study presented in this paper, its level varied according to the type of infant formula. The highest level of this acid was recorded in IF-VII and the lowest in IF-V. According to the manufacturer’s data on the label, one of the fat sources in IF-VII was high-oleic canola oil, which may have contributed significantly to the level of C18:1 *n*-9 acid. The oleic acid content in IF-I, IF-IV, and IF-V was the same as that determined in one of the infant formulas by Wu et al. [15]. On the other hand, Mendonça et al. [3] determined much higher amounts of oleic acid in a standard infant formula. This may be evidenced by the higher percentage of oils containing oleic acid (canola, sunflower, palm, and coconut) used in the production of infant formula. In the conducted study, of the seven infant formulas tested, IF-VI and IF-VII, with palm oil in the first place in the formulation, contained the most MUFAs. Palm oil consists of 40% MUFAs and is the richest in oleic acid [16].

#### 2.1.3. Polyunsaturated Fatty Acids (PUFAs)

Of the *n*-3 PUFAs, the levels of docosahexaenoic acid (DHA) and eicosapentaenoic acid (EPA) were at similar levels in each of the milk, except for IF-III, which had lower levels of EPA compared with the others. DHA content ranged from 0.67% in IF-II to 0.77% in IF-V and IF-VI. According to recommendations, DHA content in infant formulas should be in the range of 0.5–1% of the total content of all fatty acids [17]. On the other hand, Wu et al. [15] indicated a nearly four times lower content of DHA acid in infant formulas, concerning the results of our study. Among the seven infant formulas studied, α-linolenic acid (C18:3 *n*-3) had the highest variability in omega-3, with amounts ranging from 1.25% in IF-IV to 2.29% in IF-III and IF-VI. The higher content of C18:3 *n*-3 acid in the IF-II, IF-III, and IF-VI formulas may be due to the addition of fish oil (IF-II) and *Mortierella alpina* Peyronel oil (IF-VI, IF-VII) (according to the manufacturer’s packaging). Among PUFAs *n*-6, the content of arachidonic acid (C20:4 *n*-6) in the infant formulas tested did not differ significantly, ranging from 0.40–0.46%. Moreover, these values were the same in IF-VI and IF-VII, which contained *Mortierella alpina* Peyronel oil as one of their fat sources. In the formulation of infant formulas, fat sources derived from unicellular organisms, such as the fungi *Mortierella alpina* Peyronel or *Schizochytrium* sp., are increasingly used. *Mortierella alpina* Peyronel is an oleaginous mushroom that is used to produce oil and polyunsaturated fatty acids. In its composition, it has a particularly high content of arachidonic acid (almost 40%), as well as oleic, linoleic, and α-linolenic acids [18]. In the case of linoleic acid (C18:2 *n*-6), the highest amount was identified in IF-VI, which could be significantly influenced by the addition of *Mortierella alpina* Peyronel oil as a fat source (according to the manufacturer’s packaging). According to the 2016 European Commission (EC) Directive, the levels of LA and ALA are 10–25% and 1–2%, respectively [19]. Compared with the previous EC Directive of 2006, there is now a higher minimum addition of LA acid and lower ALA acid, which were previously 12–15% and 1.5–2.5%, respectively [9,20]. Following the current regulations, LA levels in each of the infant formulas tested were within the indicated range, while ALA levels were slightly higher in IF-II, IF-III, and IF-VI I. It should be noted, however, that other bodies (e.g., the European Food Safety Authority (EFSA) and the Food Standards Australia New Zealand (FSANZ)) indicate slightly different standards for PUFA content in infant formulas, which are set based on their expert consultations [21].

#### 2.1.4. Trans Fatty Acids (TFAs)

In the analyzed infant formulas, five acids from the TFA group were identified. The amount of individual TFAs was at very low levels (0.01–0.07%), which can be considered trace amounts. The TFAs detected in infant formulas mainly come from two sources—milk fat and vegetable oils [22]. Since TFAs are endogenous components of cow’s milk, TFAs are allowed to be present in infant formulas up to 3% of total fatty acids, according to the Codex Alimenatrius Commission of 2015 [23]. However, it should be emphasized that hydrogenated fats available on the market must not be used in the production of infant formulas [24].

### 2.2. Comparison of the Fatty Acid Profile of Infant Formulas with Breast Milk

**Figure 1:**
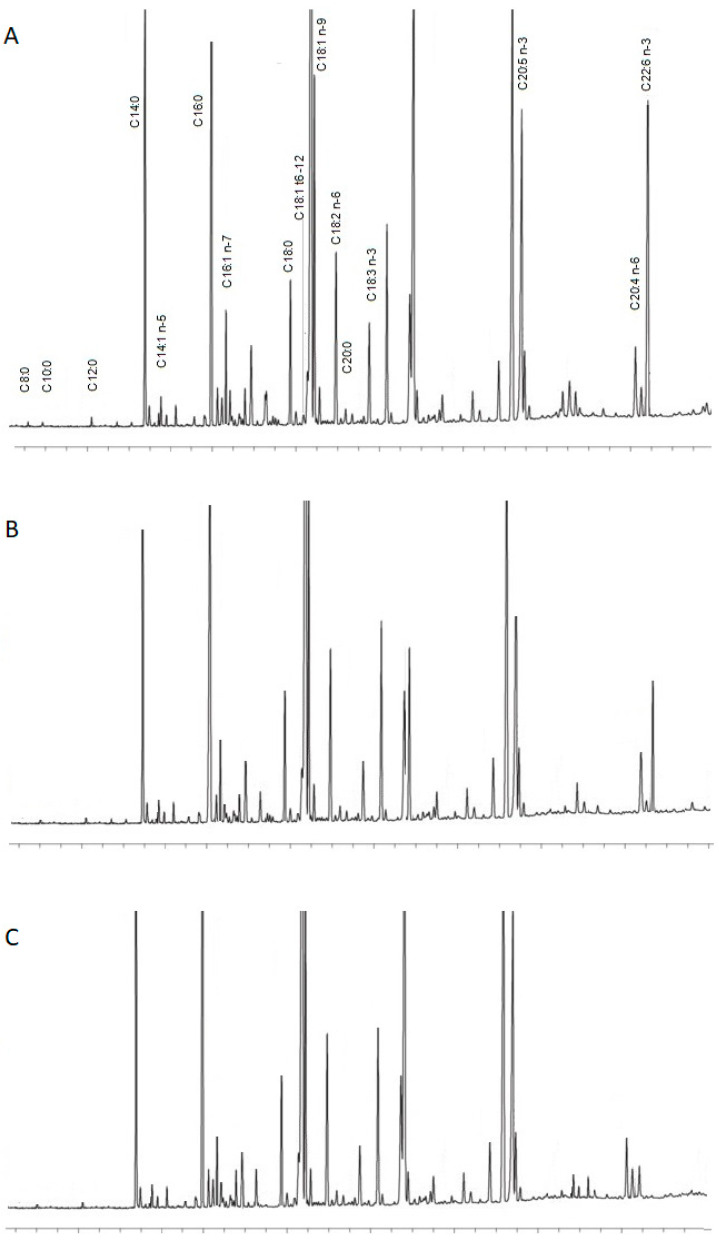
Chromatograms of separations obtained from a used standard (**A**), of a sample of infant formula (**B**), and of a sample of human milk (**C**).

Table 2 summarizes the average content of individual fatty acids from seven infant formulas and their content in mature breast milk samples. The present study found that the lipid composition of infant formulas differed significantly from that of breast milk. Figure 1 presents examples of obtained chromatograms in infant formulas and human milk.

#### 2.2.1. Saturated Fatty Acids (SFAs)

According to the literature, the SFA fraction in breast milk ranges from 28.70 to 48.99% [3]. The values obtained in the conducted study were slightly higher [49.97%], which may be explained by the high variability in the lipid fraction in breast milk. The fatty acid profile of breastmilk depends on, among other things, the race and origin of the mothers, established and used dietary habits, and also the lactation period [25]. The overall SFA content of infant formulas was similar to that of breast milk, but the proportions of individual acids differed. Zhang et al. [26] report that SFA content in breast milk varies worldwide from 35% to 54.5%, with the level in Poland at 43.6%. Lower SFA content in breast milk is reported in Turkey [30.8%], Greece [35.5%], Portugal [32.6%], and Asian countries [South Korea—35%; Thailand—34.1%; Iraq—31.2%; China—31.8%]. These countries indicate a higher consumption of fruits and vegetables and record a lower intake of animal products in their diet. In contrast, the high consumption of meat and meat products is popularized in countries such as Germany, Poland, and Hungary. The high proportion of these products in women’s diets may affect SFA levels in breast milk [25]. The consumption of cholesterol-rich products is reported to correlate with the SFA content of milk [27]. Infant formulas contained significantly more MCFAs, including more than eight times more caprylic acid and more than 40% more lauric acid. These differences are attributable to the type of fat used to produce infant formulas (coconut oil, palm oil), which contain high levels of MCFAs [15]. Very often, the addition of vegetable oils results in higher levels of SCFAs and MCFAs in infant formulas than in breast milk [7]. MCFAs are included in infant formulas for their direct absorption through the portal vein and production of a fast source of energy for infants [28]. For lauric acid content, a similar trend was observed by Sánchez-Hernández et al. [7]. Palmitic acid content was at a higher level in human milk [26.38%] than in infant formulas [21.84%] (*p* ≤ 0.05). In a study by Mendonça et al. [3], the content of this acid was lower in the mature fraction of breast milk (19.48%). Other studies indicate that the amount of palmitic acid in breast milk varies between 17.30 and 25.14% [29]. Palmitic acid has a very important function for the infant, providing better digestibility and utilization of this nutrient as an energy source. In addition, this acid can be effectively stored as an energy source by the infant [3]. The high utilization of palmitic acid is attributed to its occurrence in human milk in the *sn*-2 position, which allows pancreatic lipases to selectively hydrolyze fatty acids in the *sn*-1 and *sn*-3 positions. As a result, *sn*-2 monoacylglycerols and free fatty acids are secreted, which are then absorbed, re-esterified, and secreted into the plasma [30]. On the other hand, infant formulas have mainly vegetable oils in their composition; therefore, palmitic acid is primarily esterified at the *sn*-1 and *sn*-3 positions. Consequently, free palmitic acid binds calcium, becomes insoluble in the intestine, and is poorly absorbed and excreted with feces [31]. For the other SFAs, there were significantly more than twice as high levels of stearic acid in breast milk, similar to the study by Mendonça et al. [3]. In contrast, levels of arachidic acid were significantly higher in infant formulas than in human milk (*p* ≤ 0.05), as in Sánchez-Hernández et al. [7].

#### 2.2.2. Monounsaturated Fatty Acids (MUFAs)

Among MUFAs, there were significant differences in oleic acid content between human milk and infant formulas—breast milk contained about 20% less oleic acid (*p* ≤ 0.05). In a study by Sánchez-Hernández et al. [7] involving mothers from a region of Spain, oleic acid levels in breast milk were significantly higher. This may be related to the practice of a Mediterranean diet in this region and a much higher proportion of olive oil in the diet, in particular [12]. There are studies indicating the intake of MUFA on their content in HM. Jagodic [32] indicated a relationship between maternal seafood consumption and MUFA levels in HM. In addition, in the study by the aforementioned authors, the level of oleic acid was at the same level in maternal milk and infant formulas [7]. On the other hand, Wu et al. [15] indicated a higher level of oleic acid by more than 22% in infant formula as opposed to breast milk, which is similar to the results of our study.

#### 2.2.3. Polyunsaturated Fatty Acids (PUFAs)

Of all the PUFAs, linoleic acid (18:2 *n*-6) was the dominant one, accounting for 15.47% and 10.42% in infant formulas and breast milk, respectively. The content of this acid in infant formulas was more than 30% higher than in human mature milk (*p* ≤ 0.05). The level of linoleic acid determined in the breast milk samples studied was at a relatively low level in relation to other results from the literature [3,7,15,33]. The content of linoleic acid in infant formulas and breast milk should range from 7 to 20% of total fatty acids. This amount enables to cover the demand for this component, while higher concentrations may cause undesirable effects concerning lipoprotein metabolism and oxidative stress [3]. 

It has been reported that α-linolenic acid (ALA) [1.92%], docosahexaenoic acid (DHA) [0.73%], and arachidonic acid (AA) [0.43%] are higher in infant formulas than in breast milk [0.90%, 0.36%, and 0.38%, respectively]. As a result of the high proportion of ALA in infant formulas, PUFA levels are much higher in them than in breast milk [7]. This is mainly attributable to accepted regulations regarding the addition of PUFAs to infant formulas, which manufacturers are required to meet. In the case of human milk, it is difficult to establish reference values for selected fatty acids because of the variable composition depending on the mother’s race, her origin, and the type of fat consumed [24]. According to the Codex Alimentarius [34], the addition of AA in infant formulas should reach at least the same concentration as DHA; such a relationship was noted in the presented study. In the case of DHA, since February 2020, all infant and young child formulas must contain 20–50 mg of DHA per 100 kcal, which corresponds to about 0.5–1% of total fatty acids [18]. The infant formulas tested met this standard, and the average DHA content was 0.73% of the proportion of all fatty acids. AA and DHA can be synthesized from ALA; however, in infants, immature enzymes (desaturases and elongases) significantly limit this transformation. Therefore, it is strictly defined what addition of these acids should be added when designing infant formulas [15]. 

It should be noted that the level of PUFAs in breast milk is highly dependent on the mother’s origin, as well as her customary diet. Zhang [26] reports that in Mediterranean countries—such as Spain, Croatia, and Turkey—PUFA levels are higher [18.4–26.9%] than in Central and Eastern European and Scandinavian countries [11.9–15.5%]. This is explained by the promoted Mediterranean diet in these countries and the high proportion of fish, seafood, grains, nuts, fruits, and vegetables in this diet. The level of fatty acids in breast milk depends on the foods traditionally consumed in a country. China and Japan are known for their high consumption of vegetable oils and seafood, which leads to relatively high concentrations of PUFAs in milk. In addition, China practices traditional postnatal care practices, known as *zouyuezi*, wherein mothers are encouraged to consume increased amounts of meat and eggs. Dietary habits and practices implemented during pregnancy and postpartum significantly affect differences in lipid concentrations in breast milk [26].

#### 2.2.4. Trans Fatty Acids (TFAs)

Breast milk may contain variable amounts of TFAs. Some of them, such as vaccenic acid (C18:1 t11) and conjugated linoleic acid (CLA), occur naturally in foods (meat, milk, and milk products), and with their consumption by the mother, they pass into breast milk. A second source of TFAs is produced industrially through grilling, frying, and oil hydrogenation processes, and it is found in products such as confectionery, bakery products, and sweets [35]. Evidence from published studies indicates that the percentage of TFAs in breast milk is influenced by their proportion in the diets of breastfeeding mothers [36]. Of the TFAs, human milk contained significantly higher amounts of each acid compared with the infant formulas analyzed (*p* ≤ 0.05). The highest amounts of linoelaidic acid (C18:2 t9.12) [0.13%] and elaidic acid (C18:1 t9) [0.12%] were noted. Other authors have identified significantly higher amounts of TFA [33,37]. In one study conducted, vaccenic acid was the dominant TFA, accounting for 1.70% of total fatty acids [35], while in our study, the level of this acid was much lower, at 0.08% of total fatty acids. The level of vaccenic acid is strongly correlated with the consumption of milk and meat and their products [35,38]; however, the presented studies did not analyze the habitual diet of lactating women and did not obtain information on the frequency of consumption of specific food groups. In this case, one can only speculate that the diet of lactating women was not rich in dairy and meat products. It is reported that the common predominant TFA in breast milk is elaidic acid, which is also the most common industrially produced TFA in food [37]. An excess of TFAs from industrial sources can have harmful effects on infant development. Their high levels can interfere with the desaturation of linoleic acid and α-linolenic acid and thus can lead to lower levels of AA and DHA. On the other hand, natural trans fatty acids found in breast milk are often associated with a lower risk of atopic dermatitis, eczema, and the occurrence of food allergies in infants. Acids such as vaccenic or conjugated linoleic acid modulate the human immune system by reducing the production of pro-inflammatory mediators—cytokines or prostaglandins [39]. In the conducted study, the total TFA content was 0.52%, which was lower than that determined in the milk of women from Canada [1.9%] [40], Brazil [2.23%] [41], China [0.77%] [13] and United States [1.09%] [42]. The high variation in TFA content in the human milk of mothers from different countries is related to the varying consumption of foods rich in these acids. According to a systematic review by Wanders et al. [43], higher amounts of TFA are consumed in the United States, Canada, and Brazil compared with Poland. A diet containing hydrogenated fats significantly affects TFA levels in breast milk, especially the C18:1 t9 content [27].

### 2.3. Lipid Quality Indices in Infant Formulas and Human Milk

Table 3 summarizes the results of the five lipid quality indices—DFA, OFA, AI, TI, and H/H—for individual infant formulas and breast milk samples.

#### 2.3.1. Index of Desirable Fatty Acids (DFAs) and Index of Index of Hypercholesterolemic Fatty Acids (OFAs)

The desirable fatty acid (DFA) index is defined as the sum of MUFA, PUFA, and stearic acid [11]. DFA values differed between infant formulas and breast milk. In infant formulas, the DFA index ranged from 57.40 to 63.72. The highest DFA levels were indicated in IF-VI and IF-VII (63.72 and 63.57, respectively). The indicated infant formulas contained fish oil, which could significantly increase the proportion of PUFA *n*-3 (ALA, EPA, and DHA acid) [44]. The obtained values of the DFA index in infant formulas were similar to those obtained by other authors: Wu et al. [15] [64.59 and 72.23], Chen et al. [28] [59.98, 65.07, 63.08, 65.95], and Yu et al. [45] [68.56]. It should be noted that DFA ratios are similar in all infant formulas studied due to regulations on infant formula preparation. In breast milk, DFA levels were significantly lower [51.68] than those in infant formulas (*p* ≤ 0.05). This is primarily due to the lower proportion of LA and ALA acid in the breast milk studied. Against the background of available data in the literature, the level of DFA in the milk of the studied women was relatively low. The lowest assessed DFA index was that in the milk of lactating women from Kenya [54.19] [14] and Bahrain [57.67] [46]. DFA levels were slightly higher in milk from women from Brazil [63.44] [47], Canada [66.79] [25], and China [70.88] [48]. Zielińska-Pukos et al. [49] emphasize that the level of PUFAs in breast milk is largely influenced by women’s habitual diet and the proportion of foods rich in selected acids (LA, ALA, DHA, ARA). 

It is indicated that lauric (C12:0) and myristic (C14:0) acids have the effect of increasing total cholesterol compared with other fatty acids [50]. The percentage of fatty acids with hypercholesterolemic (OFA) potential, which corresponds to the sum of lauric (C12:0), myristic (C14:0), and palmitic (C16:0) acids, ranged from 33.36 to 38.06 in infant formulas. Similar percentages of these acids in infant formulas were obtained by other authors [15,51]. Slightly lower levels of these acids were obtained by Mendonça et al. [3] [26.08] and Korma et al. [52] [25.56]. In the studies conducted, the OFA rate in breast milk slightly exceeded that determined for infant formulas. Comparing this result with the outcomes from the literature, similar results of the OFA index in breast milk have been observed [45,53]; however, some works have indicated its different levels [26,54,55]. It should be taken into account that the fatty acid composition of breast milk depends on many factors and changes depending on the place of origin of the mothers, the time of day, and also the recent food intake [7]. Sinanoglou et al. [56] reported that more SFAs, especially lauric acid and myristic acid, are present in the milk of women under the age of 35. Additionally, according to literature reports, the higher presence of these acids in breast milk fat is due to a high-carbohydrate diet [57]. It should also be noted that the presence of lauric and myristic acid in breast milk fat, which has potentially atherogenic effects, can be compensated for by the numerous antimicrobial and antiviral properties of these acids [26]. For infant formulas, it is recommended that the sum of lauric and myristic acids should not exceed 20% [7], which, in the studies conducted, does not exceed the value of 17.89%.

#### 2.3.2. Index of Atherogenicity (AI), Index of Thrombogenicity (TI), and Hypocholesterolemic/Hypercholesterolemic (H/H) Ratio

AI values for infant formulas ranged from 0.49 to 0.98, while in breast milk, the value was 1.47. Similar AI values in breast milk were obtained by Sinanoglou et al. [56] and Pietrzak-Fiećko and Kamelska-Sadowska [58]. In turn, the values of the TI index in infant formulas ranged from 0.48 in IF-III to 0.60 in IF-IV, while in breast milk, its value was 1.60 and significantly higher (*p* ≤ 0.05). AI and TI take into account the different effects of individual fatty acids on the likelihood of diseases such as atherosclerosis and ischemic heart disease. The AI value indicates the relationship between the sum of major SFAs and the sum of major MUFA classes. In contrast, TI reports the ratio between prothrombogenic (SFA) and anti-thrombogenic (MUFA, PUFA *n*-3 and *n*-6) fatty acids [59]. Although the lipid requirements of infants are different from those of adults, AI and TI values in infant milk are at low levels [56] compared with the milk of other mammals. Pietrzak-Fiećko and Kamleska-Sadowska [58] reported that the AI is 2.37, 3.17, and 4.21 for cow’s, goat’s, and sheep’s milk, respectively. 

The last indicator assessed was the H/H ratio, which describes the relationship between hypocholesterolemic fatty acids (C18:1, PUFA) and hypercholesterolemic fatty acids (C12:0, C14:0 and C16:0) [11]. In infant formulas, the H/H ratio ranged from 1.93 to 2.30. Higher values of the ratio were observed in IF-II, IF-III, and IF-VI (2.26, 2.30, and 2.23, respectively). This can be largely explained by the types of fat used in the production of each infant formula. One of the fat sources used in IF-II and IF-III was high oleic sunflower oil, which significantly affected the oleic acid levels in the products. In addition, the indicated infant formulas had fish oil (IF-II) and *Mortriella alpina* Peyronel oil in the formulation, which could significantly affect the higher values of the H/H ratio. The H/H ratio in breast milk was significantly lower than that obtained in infant formulas (*p* ≤ 0.05). It has been reported that lower values of AI and TI ratios and high H/H values characterize products with good lipid composition [60].

Breast milk is the most recommended first food for infants. Its composition is unique and adapts individually to the needs of the baby. Infants can digest human milk easily, and the ingredients it contains are well absorbed. In addition, breast milk has an important immune function and forms a unique bond between mother and child. Naturally fed babies are reported to be at a lower risk of problems with insulin resistance, diabetes, high cholesterol, and obesity in adulthood [1,2,6]. However, it should be noted that natural feeding, for many reasons, can be inconvenient, cause discomfort, and overwhelm the mother, or there may be some complications with feeding. Then, the recommended and safe method of feeding is to provide the baby with appropriate infant formulas [3]. Each infant individually responds to infant formulas; however, it has been reported that they cause digestive problems and colic more often than breast milk [61]. On the other hand, it is easier for the mother to monitor the amount of food eaten by the baby when she feeds them infant formula [62]. When choosing infant formula, it is important to carefully study the labels and select a more favorable product. It is recommended to choose infant formulas containing fish or oil, which affects the higher PUFA content in the finished product. There are also infant formulas on the market that contain triglycerides in the *sn*-2 position; their addition has a positive effect on the infant, helping to solve digestive problems and avoid colic [63]. Over the years, the composition of infant formulas, especially the lipid fraction, has improved significantly. Manufacturers have reduced the addition of palm oil and replaced it with canola and sunflower oils. Currently, work is underway to make the fat structure of infant formulas even more similar to breast milk, adding β-palmitate, naturally occurring in breast milk, as well as MFGM (Milk Fat Globule Membrane), a naturally occurring membrane that surrounds the fat droplets in breast milk, which plays an important role in brain structure and function [1,64]. These changes will certainly allow infant formulas to become even more similar to breast milk. Infant formulas, despite constant research and evaluative component changes, will never take the form of breast milk, which is always the most valuable for a baby [1,6].

### 2.4. Associations between Obtained Data—Multivariate Analysis

#### 2.4.1. Principal Component Analysis (PCA)

**Figure 2:**
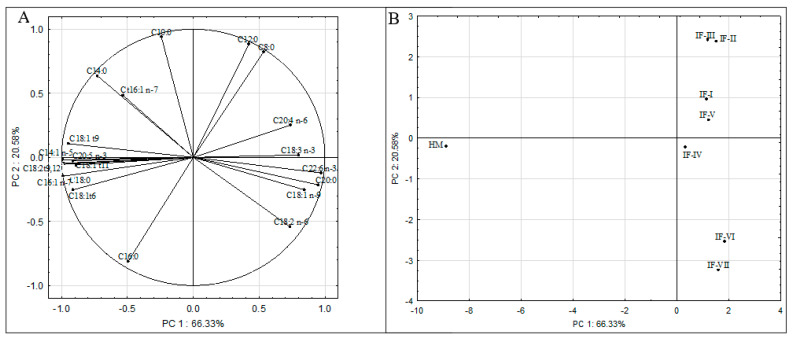
Principal component plot, variations in the selected fatty acids (C8:0, C10:0, C12:0, C14:0, C16:0, C18:0, C20:0, C14:1 *n*-5, C16:1 *n*-7, C18:1 *n*-9, C18:3 *n*-3, C20:5 *n*-3, C22:6 *n*-3, C18:2 *n*-6, C20:4 *n*-6, C18:1 t6, C18:1 t9, C18:1 t11, C18:2 t9,12, C16:1 t9) of the analyzed infant formulas and human milk samples (**A**), and score plot of the analyzed infant formulas and human milk samples (**B**). Explanations: IF-I, IF-II, IF-III, IF-IV, IF-V, IF-VI, IF-VII—infant formulas from different manufacturers; HM—human milk.

Principal Component Analysis was used to explain the structure of variation in the fatty acid composition of infant formulas and human milk samples. PCA was performed on all the tested infant formulas and human milk samples and the variables, which were the individual fatty acids (C8: 0, C10:0, C12:0, C14:0, C16:0, C18:0, C20:0, C14:1 *n*-5, C16:1 *n*-7, C18:1 *n*-9, C18:3 *n*-3, C20:5 *n*-3, C22:6 *n*-3, C18:2 *n*-6, C20:4 *n*-6, C18:1 t6, C18:1 t9, C18:1 t11, C18:2 t9,12, C16:1 t9). The two principal components described 86.91% of the variance. Figure 2A shows the relationships between the individual fatty acids and the resulting principal components. Some of the variables were transferred at a higher degree by PC1 (C14:0, C18:0, C20:0, C14:1 *n*-5, C16:1 *n*-7, C18:1 *n*-9, C18:3 *n*-3, C20:5 *n*-3, C22:6 *n*-3, C18:2 *n*-6, C20: 4 *n*-6, C18:1 t6, C18: t9, C18:1 t11, C18:2 t9,12 and C16:1 t9), while the others (C8:0, C10:0, C12:0, and C16:0) were largely transferred by PC2. Based on PCA, a positive correlation could be inferred between C8:0 and C12:0, C20:4 *n*-6 and C18:3 *n*-3, C22:6 *n*-3 and C18:3 *n*-3, and C18:1 *n*-9 and C18:2 *n*-6. In addition, individual TFAs were positively correlated with each other. A negative correlation between *n*-3 and *n*-6 group acids with TFAs was noted.

Figure 2B shows a plot of points in the plane of the principal components, which shows the similarities and differences between the fatty acid profiles of the infant formulas and human milk samples. The arrangement of the analyzed cases in relation to each other shows the different fatty acid profiles between the infant formulas studied and between infant formulas and human milk. The graph shows that IF-II and IF-III, IF-I and IF-V, and IF-VI and IF-VII have the most similar fatty acid profiles. In contrast, in terms of fat composition, IF-VI and IF-VII differ the most from IF-II and IF-III. It was clearly observed that HM was separated from all IFs, indicating that human milk samples had a different fatty acid profile than infant formula. 

The distribution of cases on the score plot is attributable to the different fatty acid profiles in infant formulas and human milk. Based on PCA, the separated HM from all IFs was the result of the increased content of TFA (C18:1 t6, C18:1 t9, C18:1 t11, C18:2 t9,12, C16:1 t9), MUFA (C14:1 *n*-5, C16:1 *n*-7), and stearic acid (C18:0) in human milk relative to infant formulas. The separated IF-VI and IF-VII relative to the other infant formulas is due to their increased content of C18:1 *n*-9 and C18:2 *n*-6. In contrast, the grouping of IF-II and IF-III at the top of the score plot is determined by their higher content of C8:0 and C12:0.

#### 2.4.2. Cluster Analysis (CA)

**Figure 3:**
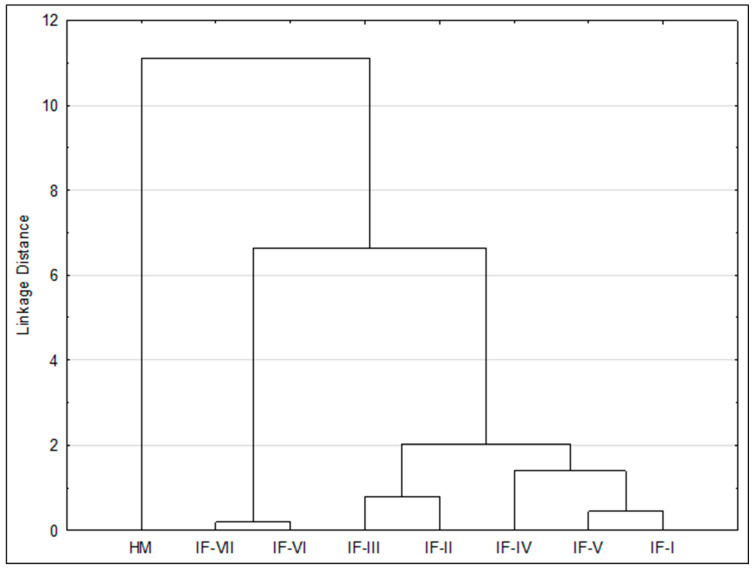
Cluster analysis (CA) of lipid quality indices in studied infant formulas and human milk. Abbreviations: IF-I, IF-II, IF-III, IF-IV, IF-V, IF-VI, IF-VII—infant formulas of selected producers, HM—human milk.

To explain the relationship between the lipid quality indices of the tested infant formulas and human milk, cluster analysis (CA) was used. To assess the variation in lipid quality indices, a cluster dendrogram was constructed based on the values of desirable fatty acids (DFAs), hypercholesterolemic fatty acids (OFAs), Index of Atherogenicity (AI), Index of Thrombogenicity (TI), and hypocholesterolemic/hypercholesterolemic (H/H) ratio. The dendrogram was determined using the weighted pair-group average method, and distances were measured using the Euclidean distance formula method. CA connects similar objects into clusters, which are represented by the horizontal axis. The vertical scale on the dendrogram represents distance or dissimilarity. When the distance between two objects is small, they are considered to be in the same cluster. Objects in the same cluster are considered to be similar in groups. The values of lipid quality indices in the studied infant formulas and human milk were divided into two main clusters (Figure 3), where the first consisted of HM and the second consisted of IF-VII, IF-VI, IF-III, IF-II, IF-IV, IF-V, and IF-I. The connections of the dendrogram showed the grouping of the studied samples based on their similarities. Cluster analysis showed that lipid quality indices were most similar in IF-I and IF-V, IF-II and IF-III, and IF-VI and IF-VII. Of the infant formulas, IF-VI and IF-VII were the most diverse in terms of lipid quality indices. Based on the dendrogram, it could be indicated that HM had significantly different lipid quality indices for all seven infant formulas. 

The distribution of clusters shown in the figure is the result of the different fatty acid content of infant formulas and human milk, which affect the values of lipid quality indices. Human milk formed a separate cluster, as it had significantly lower DFA and H/H and significantly higher OFA, AI, and TI values. These levels were determined by the lower content of MUFA and PUFA (C18:1 *n*-9, C18:2 *n*-6, C18:3 *n*-3, C22:6 *n*-3) and higher content of SFA (C14:0 and C16:0) in human milk compared with infant formulas. In cluster two, it was clear that IF-VI and IF-VII were distant from the other infant formulas, which is mainly explained by their lower SFA and higher PUFA content, which affect the values of lipid quality indices. Based on the analyses, it was indicated that IF-VI and IF-VII have significantly higher DFA contents and lower OFA and AI values relative to IF-I, IF-II, IF-III, IF-IV, and IF-V.

Both multivariate analyses, PCA and CA, indicated that infant formulas and human milk samples were characterized by different proportions of individual fatty acids. The analyses performed indicated that the distinct fatty acid profile and different lipid quality indices in breast milk as compared to infant formulas are influenced by a higher content of TFA (C18:1 t6, C18:1 t9, C18:1 t11, C18:2 t9,12, C16:1 t9)) and SFA (C14:0 and C16:0) and a lower content of MUFA (C14:1 *n*-5, C16:1 *n*-7) and PUFA (C18:2 *n*-6, C18:3 *n*-3, C22:6 *n*-3). Multivariate analyses also confirmed that IF-VI and IF-VII differed from the other infant formulas, particularly in terms of lower SFA content (C8:0, C10:0, C12:0, C14:0), higher PUFA content (C18:2 *n*-6), and, for IF-VII, higher MUFA content (C18:1 *n*-9). 

### 2.5. Limitations and Future Directions

The present study has some limitations. This study did not consider the usual diet of lactating women; therefore, it was not possible to assess the variation in the fatty acid profile against other literature results. According to the literature, the intake of selected foods significantly affects the fatty acid profile of breast milk, particularly the levels of PUFA and TFA. There were also some limitations to milk collection in this study. Because the children were fed on demand by their mothers, milk samples were not collected at regular times. To obtain representative results, it is recommended to collect milk samples at regular periods. However, during this study, importance was given to the comfort of mother and child, and no modifications were made to the method or frequency of feeding. Accordingly, milk samples were collected immediately after feeding. This procedure was followed in order not to interfere with normal infant feeding and to ensure a sufficient supply of milk. 

The present study evaluated the fat profile of seven available follow-on infant formulas and compared them to values obtained for breast milk; therefore, information on current food intake would be necessary for a full comparison. It should be emphasized that the authors analyzed only seven follow-on infant formulas; thus, the results obtained still cannot be considered entirely representative and universal. The results of this study encourage the authors to continue research involving a thorough analysis of the food intake of lactating women and a reliable assessment of the effect of habitual diet on breast milk lipidome. In addition, a good prospect for future research would be to include more infant formulas due to the constantly evolving infant food market.

## 3. Materials and Methods

### 3.1. Chemicals

Potassium hydroxide, sodium sulfate, and ammonia were purchased from Sigma Chemical Co. (St. Louis, MO, USA). Methyl and ethyl alcohol, hexane, and diethyl ether were obtained from Merck (Darmstadt, Germany).

### 3.2. Milk Samples Used in the Study

Seven types of follow-on infant formulas (for infant feeding after the sixth months) (IF-I, IF-II, IF-III, IF-IV, IF-V, IF-VI, IF-VII), which were purchased on the Olsztyn market, were used as study material. The infant formulas were based on cow’s milk and took the form of powder, as well as being prepared for the analysis according to the instructions on the packaging. Each of the infant formulas used had a low degree of protein hydrolysis. Table 4 shows the ranges of nutritional values and preparation methods for each type of modified milk. The table also includes the types of oils included in the infant formulas. In addition, 30 samples of breast milk obtained from women living in the Warmian-Masurian province were used as research material. Samples of breast milk included mature milk collected between 6 and 10 months of lactation. Women recruited for the study had to meet the following inclusion criteria: no chronic diseases, no contraindications to breastfeeding, and normal labor/birth. The collection of milk samples was preceded by the completion of a questionnaire containing information about the woman (age, place of residence, weight, height, medications recently taken) and details of pregnancy and lactation (type of delivery, lactation period, weight and height of the child, number of deliveries). Detailed characteristics of the women studied are provided in Tables S1 and S2. Each participant signed a voluntary consent to take part in the study. 

The women were asked to collect milk samples of 50–100 mL into pre-prepared sterile glass bottles using an electric breast pump immediately after the baby’s feeding. The participants were informed about the proper storage of the milk samples (4 ± 1 °C) and possible refilling to the desired volume in the next feeding. The milk samples collected by the participants were stored at −30 °C until the analytical tests were performed. Fifty-three women aged 21–33 took part in this study. Twelve of them had not provided an adequate amount of milk and were consequently excluded from the study. Of the 41 milk samples, colostrum and transitional milk samples were excluded, obtaining 30 mature milk samples.

### 3.3. Fat Extraction

Infant formula samples were prepared according to the instructions on the manufacturer’s packaging by mixing in the appropriate amount of powder and water. Samples of breast milk were thawed and thoroughly mixed, then heated to 40 ± 1 °C. The fat in the infant formula and breast milk samples was extracted using the Rose–Gottlieb method [65]. Appropriate amounts of 10% ammonia (2 mL) and ethanol (10 mL) were added to the milk sample (10 g), mixing thoroughly each time. A mixture of two solvents, diethyl ether and hexane (25 mL each), was used for fat extraction. During the extraction process, the top organic layer was collected and filtered using anhydrous sodium sulfate. The organic solvents were evaporated with a vacuum evaporator. The extraction process was performed twice.

### 3.4. Fatty Acid Methyl Ester (FAME) Preparation and Gas Chromatography (GC) Analysis

The fatty acid methyl esters (FAMEs) were prepared according to the International Dairy Federation method (IDF 182:2002: ISO 15884:2002, Milkfat: Preparation of fatty acid methyl esters). *n*-hexane and 2M KOH in methanol were added to the fat sample and the mixture was shaken. Then, the sodium hydrogen sulfate (NaHSO_4_) was added, and the mixture was centrifuged (3000 min^−1^) [66]. Chromatographic separation producing FAMEs was carried out using the gas chromatography (GC) technique using a Hewlett-Packard 6890 chromatograph with a flame ionization detector (FID) and a capillary column Supelcowax 10 (length—100 m, inner diameter—0.25 mm, liquid phase—Supelcowax 30, film thickness—0.25 mm; temperature: detector = 250 °C, dispenser = 230 °C, column = 195 °C; carrier gas—helium, flow rate—1.5 mL/min). The identification of fatty acids was performed on the basis of their retention times in relation to the retention times of FAME standards (Supelco 37 Component FAME mixture (10 mg/mL in methylene chloride)). Chemostation software (Agilent, Alpharetta, GA, USA) was used to obtain the percentage of fatty acids, and the amount of fatty acids was expressed as the weight percentage of all FAMEs [67].

### 3.5. The Lipid Quality Indices

Lipid quality indices were calculated based on equations cited by Paszczyk and Tońska [11] and Pietrzak-Fiećko and Kamelska-Sadowska [58]:

(1) Index of Desirable Fatty Acids (DFA) DFA = UFA + C18:0

UFA—unsaturated fatty acids (MUFA + PUFA)

(2) Index of Hypercholesterolemic Fatty Acids (OFA)

OFA = C12:0 + C14:0 + C16:0

(3) Index of Atherogenicity (AI)

AI = (C12:0 + (4 × C14:0) + C16:0)/((*n* − 3) PUFAs + (*n* − 6) PUFAs + MUFAs)

(4) Index of Thrombogenicity (TI)

TI = (C14:0 + C16:0 + C18:0)/((0.5 × C18:1) + (0.5 × sum of other MUFAs) + 0.5 ×

(*n* − 6) PUFAs) + (3 × (*n* − 3) PUFAs) + (*n* − 3) PUFAs/(*n* − 6) PUFAs

(5) hypocholesterolemic/hypercholesterolemic (H/H) ratio

H/H = (C18:1(*n* − 9) + C18:2(*n* − 6) + C18:3(*n* − 3))/(C12:0 + C14:0 + C16:0)

### 3.6. Statistical Analysis

Results were presented as mean ± standard deviation (SD) (fatty acid profile) or as mean (lipid quality indices—DFA, OFA, AI, TI, H/H). The distributions of the variables studied were assessed using the Shapiro–Wilk test. Meanwhile, the homogeneity of variances was tested using Levene’s test. The studied distribution was not normal, and the variance was not homogeneous; hence, non-parametric tests were used to perform the analyses. The comparisons of the collected quantitative data on differences in the content of selected fatty acids and lipid quality indices in breast milk and infant formulas were carried out using Kruskal–Wallis and Dunn’s non-parametric post hoc tests. The multivariate statistical methods Principal Component Analysis (PCA) and cluster analysis (CA) were used to evaluate the relations between the fatty acid profile and lipid quality indices of the tested infant formulas and human milk. The significance level was set at *p* ≤ 0.05. The analysis was carried out using Statistica 13.1 software (Statsoft Inc., Tulsa, OH, USA).

## 4. Conclusions

The present study evaluated the fatty acid profile and lipid quality indices of commercially available infant formulas and compared them with breast milk samples. Mother’s milk is the recommended food for infants for its unique composition, which meets the nutritional needs of the child. On the other hand, infant formulas are an alternative to natural food in case of inability to breastfeed. It was observed that the content of individual fatty acids in infant formulas varied, but each result corresponded to the standards available in the literature. The differences in the fatty acid content of infant formulas are attributable to the sources of fats used in their production. Infant formulas enriched in high-oleic sunflower oil had a higher hypocholesterolemic/hypercholesterolemic (H/H) ratio, while those with fish oil contained a higher content of *n*-3 polyunsaturated fatty acids (PUFAs *n*-3). The lipid profile and lipid quality indices of human milk samples differed significantly from infant formulas, as confirmed by Principal Component Analysis and cluster analysis. Compared with breast milk, infant formulas contained more medium-chain fatty acids, including caprylic and lauric acids, as well as higher amounts of monounsaturated fatty acids (MUFAs) and polyunsaturated fatty acids (PUFAs), with 20%, more than 30%, and more than 50% more oleic, linoleic, and α-linolenic acid, respectively. Infant formulas contained trace amounts of trans fatty acids (TFAs), while their level in breast milk was at 0.52% of total fatty acids. Infant formulas had more favorable values for lipid quality indices, including desirable fatty acids (DFAs), hypercholesterolemic fatty acids (OFAs), AI (Index of Atherogenicity), Index of Thrombogenicity (TI), and hypocholesterolemic/hypercholesterolemic (H/H) ratio. The composition of infant formulas has improved significantly over the years, and manufacturers are trying to bring their composition closer to breast milk, especially with the addition of docosahexaenoic acids and acids in the *sn*-2 position. Although infant formulas are made to mimic breast milk as closely as possible, it is difficult to compare the two types of food due to the high variability in the lipid fraction of human milk. The lipid composition of breast milk varies depending on the lactation period, time of day, the mother’s origin, and the type of food consumed. For this purpose, it would be necessary to study more factors that affect lipid levels in breast milk, including women’s anthropometric parameters, lactation period, and habitual food intake. The conducted research provides a future perspective for further work on the lipidome of infant foods. The authors intend to include more diverse types of infant formulas in future studies and also to evaluate the influence of individual factors on the fatty acid profile of breast milk, particularly the habitual diet of lactating women. 

## Figures and Tables

**Table 1:** Content of saturated fatty acids (SFAs), monounsaturated fatty acids (MUFAs), polyunsaturated *n*-3 and *n*-6 fatty acids (PUFAs *n*-3 and *n*-6) and trans fatty acids (TFAs) in the tested infant formulas (%).

Fatty Acid	IF-I	IF-II	IF-III	IF-IV	IF-V	IF-VI	IF-VII
Caprylic (C8:0)	1.96 ± 0.21 ^b^	2.41 ± 0.11 ^a^	2.40 ± 0.14 ^a^	1.82 ± 0.07 ^b^	1.92 ± 0.11 ^b^	0.59 ± 0.04 ^c^	0.56 ± 0.01 ^c^
Capric (C10:0)	1.52 ± 0.16 ^b^	1.58 ± 0.09 ^b^	1.77 ± 0.10 ^a^	1.39 ± 0.05 ^b^	1.45 ± 0.08 ^b^	0.57 ± 0.03 ^c^	0.59 ± 0.01 ^d^
Lauric (C12:0)	10.68 ± 1.33 ^b^	11.79 ± 0.44 ^a^	12.86 ± 0.73 ^a^	10.18 ± 0.29 ^b^	10.82 ± 0.80 ^b^	6.98 ± 0.32 ^c^	6.40 ± 0.12 ^d^
Myristic (C14:0)	4.61 ± 0.43 ^b^	5.25 ± 0.14 ^a^	5.33 ± 0.21 ^a^	4.52 ± 0.18 ^b^	4.87 ± 0.32 ^b^	2.94 ± 0.09 ^d^	0.87 ± 0.06 ^d^
Palmitic (C16:0)	22.33 ± 0.21 ^b^	18.21 ± 0.91 ^c^	17.85 ± 0.77 ^c^	22.53 ± 0.46 ^b^	22.37 ± 0.38 ^b^	23.44 ± 0.17 ^b^	26.17 ± 0.95 ^a^
Stearic (C18:0)	3.41 ± 0.07 ^b^	3.25 ± 0.14 ^c^	3.16 ± 0.07 ^c^	3.63 ± 0.09 ^a^	3.41 ± 0.09 ^b^	3.40 ± 0.08 ^b^	3.72 ± 0.09 ^a^
Arachidic (C20:0)	0.33 ± 0.02 ^b^	0.33 ± 0.01 ^b^	0.33 ± 0.01 ^b^	0.28 ± 0.01 ^c^	0.32 ± 0.01 ^b^	0.36 ± 0.01 ^b^	0.41 ± 0.03 ^a^
Myristoleic (C14:1 *n*-5)	0.01 ± 0.00 ^a^	0.02 ± 0.01 ^a^	0.02 ± 0.00 ^a^	0.02 ± 0.00 ^a^	0.02 ± 0.00 ^a^	0.01 ± 0.00 ^a^	0.02 ± 0.00 ^a^
Palmitoleic (C16:1 *n*-7)	0.18 ± 0.02 ^a^	0.17 ± 0.02 ^b^	0.18 ± 0.02 ^a^	0.17 ± 0.01 ^b^	0.18 ± 0.02 ^a^	0.18 ± 0.00 ^a^	0.21 ± 0.03 ^a^
Oleic (C18:1 *n*-9)	35.27 ± 1.56 ^c^	37.82 ± 0.67 ^b^	38.03 ± 0.24 ^b^	35.56 ± 0.402 ^c^	35.14 ± 0.70 ^c^	38.74 ± 0.31 ^b^	40.10 ± 0.78 ^a^
α-Linolenic (C18:3 *n*-3)	1.80 ± 0.11 ^c^	2.02 ± 0.04 ^a^	2.29 ± 0.10 ^a^	1.25 ± 0.03 ^d^	1.77 ± 0.08 ^c^	2.29 ± 0.15 ^a^	1.99 ± 0.15 ^c^
Eicosapentaenoic (EPA) (C20:5 *n*-3)	0.08 ± 0.01 ^a^	0.09 ± 0.03 ^a^	0.05 ± 0.01 ^b^	0.09 ± 0.02 ^a^	0.07 ± 0.01 ^a^	0.07 ± 0.01 ^a^	0.07 ± 0.01 ^a^
Docosahexaenoic (DHA) (C22:6 *n*-3)	0.70 ± 0.12 ^a^	0.67 ± 0.06 ^a^	0.72 ± 0.11 ^a^	0.71 ± 0.12 ^a^	0.77 ± 0.12 ^a^	0.77 ± 0.12 ^a^	0.74 ± 0.09 ^a^
Linoleic (C18:2 *n*-6)	15.51 ± 0.25 ^c^	13.29 ± 0.31 ^c^	13.07 ± 1.04 ^c^	16.77 ± 0.17 ^b^	15.54 ± 0.28 ^c^	17.84 ± 0.25 ^a^	16.29 ± 0.92 ^b^
Arachidonic (C20:4 *n*-6)	0.43 ± 0.04 ^a^	0.46 ± 0.07 ^a^	0.42 ± 0.03 ^a^	0.40 ± 0.04 ^a^	0.42 ± 0.04 ^a^	0.42 ± 0.04 ^a^	0.43 ± 0.05 ^a^
Petroselaidic (C18:1t6)	0.04 ± 0.00 ^a^	0.04 ± 0.00 ^a^	0.05 ± 0.01 ^a^	0.06 ± 0.01 ^a^	0.03 ± 0.02 ^b^	0.05 ± 0.02 ^a^	0.06 ± 0.00 ^a^
Elaidic (C18:1 t9)	0.02 ± 0.00 ^b^	0.04 ± 0.00 ^a^	0.05 ± 0.01 ^a^	0.03 ± 0.00 ^b^	0.04 ± 0.01 ^a^	0.03 ± 0.01 ^b^	0.03 ± 0.01 ^b^
Vaccenic (C18:1 t11)	0.01 ± 0.00 ^a^	ND	0.04 ± 0.01 ^a^	0.02 ± 0.00 ^b^	0.02 ± 0.00 ^b^	0.02 ± 0.00 ^b^	0.02 ± 0.00 ^b^
Linoelaidic (C18:2t9,12)	ND	ND	0.01 ± 0.00 ^a^	ND	0.01 ± 0.00 ^a^	ND	0.01 ± 0.00 ^a^
Palmitelaidic (C16:1 t9)	0.07 ± 0.00 ^a^	0.07 ± 0.01 ^a^	0.06 ± 0.01 ^a^	0.03 ± 0.01 ^b^	0.03 ± 0.01 ^b^	0.04 ± 0.01 ^b^	0.04 ± 0.02 ^b^
∑ SFA	44.84 ^b^	42.82 ^d^	43.70 ^c^	44.35 ^b^	45.16 ^a^	38.28 ^e^	38.72 ^e^
∑ MUFA	35.46 ^d^	38.01 ^c^	38.23 ^c^	35.75 ^d^	35.34 ^d^	38.93 ^b^	40.33 ^a^
∑ PUFA *n*-3	2.58 ^c^	2.78 ^b^	3.06 ^a^	2.05 ^d^	2.61 ^c^	3.13 ^a^	2.80 ^b^
∑ PUFA *n*-6	15.94 ^d^	13.75 ^e^	13.49 ^f^	17.17 ^b^	15.96 ^d^	18.26 ^a^	16.72 ^c^
Σ TFA	0.14 ^b^	0.15 ^b^	0.21 ^a^	0.14 ^b^	0.13 ^b^	0.14 ^b^	0.16 ^b^

Abbreviations: IF—infant formula; ND—non-detected; SFA—saturated fatty acid; MUFA—monounsaturated fatty acid; PUFA—polyunsaturated fatty acid; TFA—trans fatty acid. Means with different letters (a, b, c, d, e, f) are significantly different at *p* ≤ 0.05. Means a, b, c, d, e, f specify differences between fatty acid composition in selected infant formulas.

**Table 2:** The fatty acid content of infant formulas (average of seven infant formulas tested) and breast milk (%).

Fatty Acid	Mean Values of IF	HM
Caprylic (C8:0)	1.66 ± 0.49 ^a^	0.20 ± 0.09 ^b^
Capric (C10:0)	1.27 ± 0.36 ^b^	1.50 ± 0.70 ^a^
Lauric (C12:0)	9.96 ± 2.63 ^a^	6.58 ± 3.39 ^b^
Myristic (C14:0)	4.06 ± 1.06 ^b^	8.14 ± 2.57 ^a^
Palmitic (C16:0)	21.84 ± 4.06 ^b^	26.38 ± 6.82 ^a^
Stearic (C18:0)	3.43 ± 0.63 ^b^	7.07 ± 1.48 ^a^
Arachidic (C20:0)	0.33 ± 0.06 ^a^	0.10 ± 0.11 ^b^
Myristoleic (C14:1 *n*-5)	0.02 ± 0.01 ^b^	0.17 ± 0.08 ^a^
Palmitoleic (C16:1 *n*-7)	0.18 ± 0.04 ^b^	1.75 ± 0.67 ^a^
Oleic (C18:1 *n*-9)	37.24 ± 7.45 ^a^	30.48 ± 5.17 ^b^
α-Linolenic (ALA) (C18:3 *n*-3)	1.92 ± 0.46 ^a^	0.90 ± 0.05 ^b^
Eicosapentaenoic (EPA) (C20:5 *n*-3)	0.07 ± 0.03 ^b^	0.15 ± 0.09 ^a^
Docosahexaenoic (DHA) (C22:6 *n*-3)	0.73 ± 0.15 ^a^	0.36 ± 0.06 ^b^
Linoleic (LA) (C18:2 *n*-6)	15.47 ± 2.88 ^a^	10.42 ± 1.44 ^b^
Arachidonic (AA) (C20:4 *n*-6)	0.43 ± 0.09 ^a^	0.38 ± 0.11 ^b^
Petroselaidic acid (C18:1t6)	0.05 ± 0.01 ^b^	0.11 ± 0.02 ^a^
Elaidic acid (C18:1 t9)	0.03 ± 0.01 ^b^	0.12 ± 0.05 ^a^
Vaccenic acid (C18:1 t11)	0.02 ± 0.01 ^b^	0.08 ± 0.01 ^a^
Linoelaidic acid (C18:2t9,12)	0.01 ± 0.01 ^b^	0.13 ± 0.08 ^a^
Palmitelaidic Acid (C16:1 t9)	0.05 ± 0.02 ^a^	0.08 ± 0.06 ^a^
∑ SFA	42.55 ^b^	49.97 ^a^
∑ MUFA	37.44 ^a^	32.4 ^b^
∑ PUFA *n*-3	2.72 ^a^	1.41 ^b^
∑ PUFA *n*-6	15.90 ^a^	10.80 ^b^
Σ TFA	0.16 ^b^	0.52 ^a^

Abbreviations: IF—infant formula; HM—human milk; SFA—saturated fatty acid; MUFA—monounsaturated fatty acid; PUFA—polyunsaturated fatty acid; TFA—trans fatty acid. Means with different letters (a, b) are significantly different at *p* ≤ 0.05. Means a, b specify differences between fatty acid composition in infant formulas and human milk.

**Table 3:** Lipid quality indices (DFA, OFA, AI, TI, H/H) in infant formulas and human milk.

Lipid Quality Indices	IF-I	IF-II	IF-III	IF-IV	IF-V	IF-VI	IF-VII	HM
DFA	57.40 ^b^	57.79 ^b^	57.94 ^b^	58.60 ^b^	57.32 ^b^	63.72 ^a^	63.57 ^a^	51.68 ^c^
OFA	37.62 ^b^	35.25 ^c^	36.04 ^c^	37.23 ^b^	38.06 ^b^	33.36 ^d^	33.44 ^d^	41.10 ^a^
AI	0.95 ^b^	0.94 ^b^	0.95 ^b^	0.92 ^b^	0.98 ^b^	0.70 ^c^	0.49 ^d^	1.47 ^a^
TI	0.59 ^b^	0.49 ^d^	0.48 ^d^	0.60 ^b^	0.59 ^b^	0.51 ^c^	0.53 ^c^	1.60 ^a^
H/H	1.95 ^c^	2.26 ^a^	2.30 ^a^	1.98 ^c^	1.93 ^c^	2.23 ^a^	2.19 ^b^	1.21 ^d^

Abbreviations: IF—infant formula; HM—human milk; DFA—desirable fatty acid, OFA—hypercholesterolemic fatty acid; AI—Index of Atherogenicity; TI—Index of Thrombogenicity; –H/H—hypocholesterolemic/hypercholesterolemic ratio. Means with different letters (a, b, c, d) are significantly different at *p* ≤ 0.05. Means a, b, c, d specify differences in the level of lipid quality indices between selected infant formulas and human milk.

**Table 4:** The average content of selected nutrients in infant formulas used in this study, the method of preparation of infant formulas, and the types of oils added to their production.

Nutritional Value in 100 mL of Ready-to-Use Product *	Range of Tested Infant Formulas
Energy (kcal)	65–68
Fat (g), of which	3.0–3.7
Saturated fatty acids (g)	0.8–1.6
Monounsaturated fatty acids (g)	1.4–1.6
Polyunsaturated fatty acids (g)	0.5–0.7
Linoleic acid, LA (mg)	420–621
α-linolenic acid, ALA (mg)	39–60
Docosahexaenoic acid, DHA (mg)	11.75–17.00
Carbohydrates (g), of which	7.20–8.45
Sugars (g)	4.6–8.3
Protein (g)	1.29–2.15
Preparation of 100 mL of milk	13.05–14.10 g of powder + 90 mL of water
Oils used in the production of the tested infant formulas	Sunflower; rapeseed; coconut; palm; high oleic sunflower; coconut; high *sn*-2 palmitic palm; high oleic sunflower; low erucic rapeseed; soybean; fish; oil extracted from unicellular organisms (*Mortierella alpina* oil, *Schizochytrium* sp. oil).

* Data were obtained from the labels of individual infant formulas.

## Data Availability

The data presented in this study are available on reasonable request from the corresponding author.

## Supplementary Materials

The following supporting information can be downloaded at https://www.mdpi.com/article/10.3390/molecules29092044/s1, Table S1: Characteristics of the women studied with age, weight and height, BMI, lactation time, and child height and weight.; Table S2: Characteristics of the women studied in terms of place of residence, lactation period, type of delivery held, and number of children born.

## List of Abbreviations

AA	arachidonic fatty acid (C20:4 *n*-6)
AI	Index of Atherogenicity
ALA	α-linolenic fatty acid (C18:3 *n*-3)
DFA	Index of Desirable Fatty Acids
DHA	docosahexaenoic acid (C22:6 *n*-3)
EPA	eicosapentaenoic acid (C20:5 *n*-3)
FAMEs	fatty acid methyl esters
H/H	hypocholesterolemic/hypercholesterolemic ratio
HM	human milk
IF	infant formula
LA	linoleic fatty acid (C18:2 *n*-6)
MCFA	medium-chain fatty acid
MUFAs	monounsaturated fatty acids
OFA	Index of Hypercholesterolemic Fatty Acids
PUFAs *n*-3	polyunsaturated fatty acids *n*-3
PUFAs *n*-6	polyunsaturated fatty acids *n*-6
SCFA	short-chain fatty acid
SFAs	saturated fatty acids
TFAs	trans fatty acids
TI	Index of Thrombogenicity
