# Supplementary material for: Determination of the Fatty Acid Profile and Lipid Quality Indices in Selected Infant Formulas
Source: Molecules. 2024 Apr 29;29(9):2044. doi: 10.3390/molecules29092044 (PMC11085564; doi:10.3390/molecules29092044)
Supplement: Supplementary file 1 [file molecules-29-02044-s001.zip › molecules-2957106-supplementary.pdf]

**Table S1.** Characteristics of the women studied with age, weight and height, BMI, lactation time, and child height and weight.

| Characterisctics | Mean | SD | Range |
|---|---|---|---|
| Age [years] | 26.6 | 3.5 | 21-33 |
| Body mass [kg] | 65.6 | 9.9 | 47-85 |
| Body height [m] | 1.64 | 0.06 | 1.51-1.75 |
| Body Mass Index (BMI) [kg/m$^2$] | 24.3 | 3.3 | 17.5-29.0 |
| Time of lactation [months] | 4.9 | 3.6 | 1-10 |
| Height of the child [cm] | 53.0 | 1.9 | 51-56 |
| Weight of the child [g] | 3288.9 | 615.7 | 2400-5100 |

**Table S2.** Characteristics of the women studied in terms of place of residence, lactation period, type of delivery held, and number of children born.

| Factors | n=53 |
|---|---|
| Place of residence | |
| City | 36 |
| Village | 17 |
| Stage of lactation | |
| I | 11 |
| II | 12 |
| III | 30 |
| Type of delivery | |
| Vaginal delivery | 39 |
| Cesarean section | 14 |
| Number of children born | |
| 1 | 33 |
| 2 | 16 |
| >2 | 4 |
